# Evaluation of Production Lots of a Rapid Point-of-Care Lateral Flow Serological Test Intended for Identification of IgM and IgG against the N-Terminal Part of the Spike Protein (S1) of SARS-CoV-2

**DOI:** 10.3390/v13061043

**Published:** 2021-05-31

**Authors:** Tove Hoffman, Linda Kolstad, Bengt Rönnberg, Åke Lundkvist

**Affiliations:** 1 Department of Medical Biochemistry and Microbiology, Zoonosis Science Center (ZSC), Uppsala University, Husargatan 3, SE-751 23 Uppsala, Sweden; tove.hoffman@medsci.uu.se (T.H.); linda.kolstad@imbim.uu.se (L.K.); bengt.ronnberg@gmail.com (B.R.); 2 Laboratory of Clinical Microbiology, Uppsala University Hospital, Dag Hammarskjölds väg 38, SE-752 37 Uppsala, Sweden

**Keywords:** POC-test, lateral flow, lot, SARS-CoV-2, S1, IgM/IgG, specificity

## Abstract

The potential of rapid point-of-care (POC) tests has been subject of doubt due to an eventual risk of production errors. The aim was therefore to evaluate the two separate production lots of a commercial POC lateral flow test, intended for the detection of IgM and IgG against the SARS-CoV-2 spike protein (S1). Control samples consisted of serum from individuals with confirmed SARS-CoV-2 infection and pre-COVID-19 negative sera gathered from a biobank. The presence of anti-S1 IgM/IgG in the sera was verified by an in-house Luminex-based serological assay (COVID-19 SIA). One hundred samples were verified as positive for anti-S1 IgG and 74 for anti-S1 IgM. Two hundred samples were verified as negative for anti-S1 IgM/IgG. For the two lots of the POC-test, the sensitivities were 93.2% and 87.8% for IgM and 93.0% and 100% for IgG. The specificities were 100% for IgM and 99.5% for IgG. The positive predictive value was 100% for IgM and 98.9% and 99.0% for IgG. The negative predictive value was 97.6% and 95.7% for IgM, and 96.6% and 100% for IgG. The evaluated POC-test is suitable to assess anti-SARS-CoV-2 S1 IgM and IgG, as a measure of previous virus exposure on an individual level. The external validation of separate lots of rapid POC-tests is encouraged to ensure high sensitivity before market introduction.

## 1. Introduction

In late 2019, a novel coronavirus causing severe acute respiratory disease was identified in Wuhan, Hubei Province, China. Within months, the disease COVID-19 (Coronavirus disease 2019), caused by the novel severe acute respiratory syndrome coronavirus-2 (SARS-CoV-2) spread to cause a global pandemic, infecting over 117 million individuals worldwide, as of March 7, 2021 [1]. Diagnostic accuracy is vital, as the disease can resemble those due to other viruses and bacteria and cause a wide range of symptoms—from asymptomatic infections to those of a mild common cold or more severe symptoms, such as acute respiratory distress syndrome or multi-organ failure [2,3]. The virus is an enveloped, single-stranded RNA virus of the *Coronaviridae* family. Coronaviruses share structural similarities and are composed of 16 non-structural proteins and four structural proteins: the transmembrane spike (S), envelope, membrane, and nucleocapsid (N) proteins. The N-terminal part of the S protein, the S1 subunit, contains the receptor-binding domain (RBD) that specifically recognizes the angiotensin-converting enzyme 2 (ACE2) receptor, which SARS-CoV-2 has been identified to bind to in order to infect the human host [4,5,6].

There is evidence that a majority of patients with past COVID-19 developed neutralizing antibodies against the virus [7]. For the detection of antibodies, there have been several available serological assays developed to identify individuals with recent and past exposure and to assess the extent of exposure in a population. In turn, this might help to decide on the application, enforcement or relaxation of containment measures. As proper neutralization tests are cumbersome, time-consuming and require biosafety level 3 laboratories, there are several available tests that address different SARS-CoV-2 specific antigens, the most common being the N protein or the S protein. In a population-based study from Spain, different seroprevalences were estimated by a chemiluminescent microparticle immunoassay (CMIA) for the detection of anti-N IgG, and a lateral flow immunochromatographic assay (LFIA) for the detection of anti-S1 IgG [3]. Whether the difference in estimated seroprevalence could be explained by the dynamic appearance of antibodies targeting the different viral proteins or whether the rapid point-of-care (POC) test did not have as good performance as the CMIA-test, is unknown.

Available serological methods to date either rely on quantitative laboratory-based assays or on qualitative LFIAs. While the lateral flow tests have the advantage of being a POC-analysis where results can be given directly to the patient within minutes from sample collection, rapid POC-tests have been attributed a potential risk of production errors that may result in the unreliable performance of the test. There is, however, to our knowledge, no study published to date investigating whether the sensitivity and/or specificity varies between the production lots of a rapid test. In this study, the aim was therefore to evaluate two separate production lots of one commercially available rapid POC lateral flow test (the COVID-19 IgG/IgM Rapid Test Cassette (Zhejiang Orient Gene Biotech Co Ltd., Huzhou, China)) and its accuracy in identifying anti-S1 IgM/IgG. Furthermore, the effect of disease prevalence on positive and negative predictive values was visualized to evaluate the test´s suitability for the screening of SARS-CoV-2 antibodies in regions with varying prevalence and during different stages of the pandemic. As a reference standard, the presence of SARS-CoV-2 S1-specific IgM and IgG in positive controls were verified with an in-house Luminex-based COVID-19 assay (Magpix technology, Luminex Corporation) [8].

## 2. Materials and Methods

### 2.1. Serum Samples

Positive controls constituted of serum from Swedish COVID-19 patients or convalescents, individuals not requiring hospital care for their SARS-CoV-2 infection, confirmed positive for SARS-CoV-2 by reverse transcription real-time polymerase chain reaction (RT-qPCR) and/or serology for SARS-CoV-2-specific IgM and IgG, between April and July 2020 [8]. The positive controls were collected from 5 to 120 days post symptom debut or PCR confirmation. Negative controls constituted of serum from infants (6–14 months old) and randomly selected blood donor sera from the Uppsala Academic Hospital from individuals, without any known history of SARS-CoV-2 infection/COVID-19 and before the COVID-19 pandemic (i.e., collected 2014 and 2018 respectively).

### 2.2. Reference Method

Samples were pre-specified when evaluating the index test. The reference method used was a Luminex-based (Magpix technology, Luminex Corporation) SARS-CoV-2-specific assay (COVID-19 suspension immunoassay (COVID-19 SIA)), developed in-house and used due to high specificity and sensitivity in detecting SARS-CoV-2 S1-specific IgM and IgG [8]. Samples with confirmed SARS-CoV-2 infection that tested positive for SARS-CoV-2 S1-specific IgG with a median fluorescence intensity (MFI) ≥ 900 were used as the reference for positive IgG-samples. Of those, the samples positive for SARS-CoV-2 S1-specific IgM with an MFI ≥ 700 served as a reference for positive IgM-samples. Samples with an MFI < 300 were considered negative for anti-SARS-CoV-2 S1 IgM and IgG.

### 2.3. Index Test

The index test (rapid POC-test) used was run according to the manufacturer’s instructions (COVID-19 IgG/IgM Rapid Test Cassette; Product/Model: GCCOV-402a, Lot no. 2,003,287 (Lot A) and Lot no. 2,004,156 (Lot B); Zhejiang Orient Gene Biotech Co Ltd., Huzhou, Zhejiang, China/Healgen Scientific LLC, Houston, USA) [9]. The POC-test is based on S1 and targets anti-S1 IgM and IgG. Briefly, 5 μL of serum was added to the test slide, followed by 80 μL of the buffer provided in the kit. The results were read after 10 min (max 15 min) by the naked eye. Test results were blinded to the assessors of the index test, as well as to the assessors of the reference standard.

### 2.4. Analysis

Only index tests in which the control line changed color were regarded as valid (no test was excluded from analysis). If a line was observed for IgM and/or IgG, the test was considered positive. The intensity of the color was not judged. Sample sizes were determined in order to comply with the recommendations from the Swedish Public Health Agency for the validation of COVID-19-related serological assays [10]. The sensitivity of the index test was calculated as the proportion of index positives among reference positives, and specificity as the proportion of index negatives among reference negatives. The Wilson Score method was used to calculate 95% confidence intervals (95% CI). Analyses were performed with STATA v.13.1 (StataCorp, TX, USA). Plots for positive and negative predictive value (PPV; NPV) at different prevalence levels were created in R v.4.0.0 (R Core Team, 2020), using the ggplot2 package [11]. The reporting of results was made according to the 2015 Standards for Reporting Diagnostic Accuracy (STARD) statement [12].

## 3. Results

### 3.1. Selected Serum Samples

**Figure 1:**
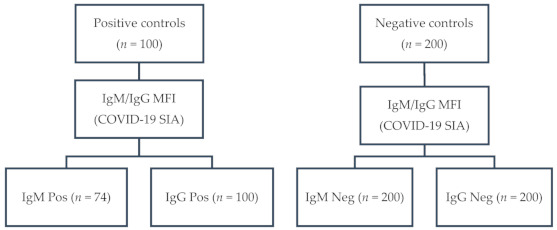
Flow diagram of sampling: IgG, immunoglobulin G; IgM, immunoglobulin M; MFI, median fluorescence intensity; COVID-19 SIA, Coronavirus disease 2019 suspension immunoassay; Pos, positive; Neg, negative.

One hundred serum samples from 52 COVID-19 patients and 48 convalescents, confirmed by RT-qPCR (*n* = 90) and/or by serology (*n* = 89), had an MFI ≥ 900 (range: 998–6477; median: 3590) for anti-S1 IgG. Of those, 74 had an MFI ≥ 700 (range: 738–5916; median: 2492) for anti-S1 IgM. None of the 200 negative control samples tested positive for anti-S1 IgM and IgG (MFI < 300). A flow diagram of the sampling is depicted in Figure 1.

### 3.2. Index Test

With the index test, none of the 200 negative sera from blood donors and infants tested IgM positive (0/200, 0% (95% CI: 0.0%–1.9%)), while one tested IgG positive (1/200, 0.5%, (95% CI: 0.1%–2.8%)). This was the case in both production lots tested (Table 1 and Table 2). The single IgG-positive sample was re-analyzed and remained IgG positive in the second test in both lots. Of the 74 IgM-positive samples, five tested IgM negative (5/74, 6.8% (95% CI: 2.9%–14.9%)) in Lot A and nine (9/74, 12.2% (95% CI: 6.5%–21.5%)) in Lot B. Of the 100 IgG-positive samples, seven tested IgG negative (7/100, 7.0% (95% CI: 3.4%–13.7%)) in Lot A while none tested IgG negative in Lot B (0/100, 0% (95% CI: 0.0% to 3.7%)). The MFI of the false IgG negatives in Lot A ranged 998–3453. The MFI of the false IgM negatives ranged 761–1340 and 738–1641 for Lot A and B, respectively.

### 3.3. Sensitivity, Specificity, and Predictive Values

**Figure 2:**
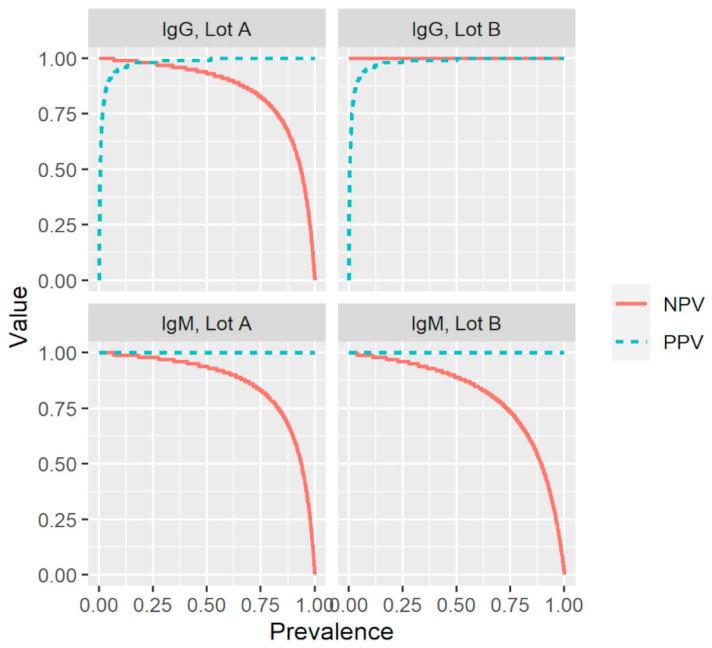
Effect of prevalence on the positive and negative predictive values (PPV; NPV) for two production lots (A and B) of a commercial rapid lateral flow point-of-care test, using the values for sensitivity and specificity presented in Table 3. IgG, immunoglobulin G; IgM, immunoglobulin M; Sens, sensitivity; Spec, specificity.

Based on the results described above and summarized in Table 1 and Table 2, the index test had a sensitivity of 87.8% and 93.2% for IgM and 93.0% and 100% for IgG (Table 3). The test exhibited an overall specificity of 100% for IgM and 99.5% for IgG. The PPV (probability of having been infected and having antibodies given a positive test result) was 100% for IgM and 98.9% and 99.0% for IgG. The NPV (probability of not yet been infected and not having antibodies given a negative test result) was 95.7% and 97.6% for IgM, and 96.6% and 100% for IgG. To evaluate the effect of disease prevalence on the reliability of the results of the index test, the positive and negative predictive values were plotted as a function of prevalence using the values for sensitivity and specificity in Table 3 (Figure 2). For both production lots and antibody types, positive and negative predictive values (PPV; NPV) remained high over a broad prevalence range.

## 4. Discussion

In this study, different production lots of a commercially available rapid POC-test for the detection of IgM and IgG against the S1-protein of SARS-CoV-2 were evaluated. The test displayed a high sensitivity and specificity when compared against a Luminex-based (Magpix technology) SARS-CoV-2-specific assay. Comparing two different production lots, the sensitivity was 93.2% and 87.8% for IgM and 93.0% and 100% for IgG. Regarding the specificity, it remained at 100% and 99.5% for IgM and IgG, respectively. A high performance of the rapid POC-test was also reported elsewhere, with a specificity ranging from 97.5% to 100% for IgG and 100% for IgM, and a sensitivity ranging from 96.7% to 98% for IgG and 68% to 100% for IgM [13]. The Swedish Public Health Agency warranted an IgG specificity of ≥ 99.5% and a sensitivity of ≥ 90% for COVID-19-related serological assays to be recommended as an assay for the assessment of antibody presence on an individual level [10]. In this study, both production lots of the investigated POC-test reached that standard and may thus be suitable for antibody screening in Sweden. Differences in the sensitivity of the two production lots in the current study might be explained by small differences in the amount of antigen added during the production of the rapid test, which could lead to a weaker qualitative response, and thus, effecting the sensitivity of the test. Different amounts, and not of varying quality, of the antigen would also explain why the test was still highly specific in both lots. As the measures of diagnostic accuracy are dependent on the disease prevalence and the sample panel/patient cohort, further evaluations should be performed as the pandemic progresses. In addition, international standard panels, including well-defined serum samples from patients with asymptomatic, mild, and severe infections will be valuable for future evaluations.

In a population-based study from Spain, more than 51,000 individuals were tested by a CMIA for the detection of anti-SARS-CoV-2 N IgG and the same rapid POC-test as investigated in the current study, targeting anti-SARS-CoV-2 S1 IgM/IgG [3]. Comparing the two tests, the authors observed different seroprevalences of SARS-CoV-2-specific IgG. Given the high performance in the detection of anti-S1 IgM and IgG observed in the current study, a possible explanation for the discrepancy could be that the median seroconversion time and the antibody peak time were observed to occur later for anti-S1 IgM/IgG, as compared to anti-N IgM/IgG [14,15]. Moreover, anti-S1 IgG levels were observed to be four times higher during convalescence than in the acute phase in about 40% of patients [15], while the measurement of only anti-N IgM/IgG have been observed to substantially underestimate the proportion of SARS-CoV-2 infections in general [16]. Thus, an inter-individual heterogeneity in antigens to which different patients develop antibodies against, or the heterogenous dynamics of antibody appearance, could be a possible explanation to the cumbersome process of inventing a single test that can identify past exposure to the virus with as close to 100% accuracy as possible, despite the world´s weighted efforts. As the current study only aimed to evaluate the performance of the rapid POC-test in detecting anti-SARS-CoV-2 S1 IgM/IgG, the non-population-based and predefined study design inherits a weakness in the sense that the POC-test investigated does not detect individuals with antibodies against the N- or other parts of the S-protein. Due to the inter-individual differences in the dynamics of these antibody subtypes, it has been suggested that a combination of antibody tests, targeting different viral proteins, may be the best strategy in order to increase sensitivity and/or specificity when screening for SARS-CoV-2-specific antibodies [3]. However, as anti-S antibodies can block the ACE2-receptor and thus prevent SARS-CoV-2 from infecting the human host [14], a positive result from the studied rapid POC-test could indicate immunity against re-infection as long as adequate antibody titers are upheld.

## 5. Conclusions

To the best of our knowledge, this is the first study to evaluate the impact of production lots on the performance of a rapid POC-test intended for the detection of SARS-CoV-2-specific anti-S1 IgM and IgG. The world is looking for serological assays that can help to decide on the relaxation of containment measures and POC-tests are optimal for such a purpose given the lower associated costs, easier implementation, and the potentially increased uptake as compared with laboratory-based assays. The fact that the PPV and NPV for IgG remained high over a broad range of prevalence indicates that the investigated rapid POC-test is suitable for the screening of SARS-CoV-2-specific IgG antibodies in regions with varying prevalence and during different stages of the pandemic, and therefore could aid in determining containment measures. The observed differences in sensitivity between the two production lots highlight the need for the external validation of each production lot before a rapid POC-test is made available on the market.

## Figures and Tables

**Table 1:** Results for the serum samples with SARS-CoV-2 specific anti-S1 immunoglobulin M (IgM), confirmed with Luminex technology, and pre-COVID-19 negative controls for two production lots of a rapid point-of-care test.

	Index Test					
	Lot A			Lot B		
Reference Method	IgM Positive	IgM Negative	Total	IgM Positive	IgM Negative	Total
IgM Positive	69	5	74	65	9	74
IgM Negative	0	200	200	0	200	200
Total	69	205	274	65	209	274

**Table 2:** Results for the serum samples with SARS-CoV-2 specific anti-S1 immunoglobulin G (IgG), confirmed by Luminex technology, and pre-COVID-19 negative controls for two production lots of a rapid point-of-care test.

	Index Test					
	Lot A			Lot B		
Reference Method	IgG Positive	IgG Negative	Total	IgG Positive	IgG Negative	Total
IgG Positive	93	7	100	100	0	100
IgG Negative	1	199	200	1	199	200
Total	94	206	300	101	199	300

**Table 3:** The performance of two production lots of a rapid IgM/IgG test cassette, evaluated using reference samples confirmed by Luminex technology and calculated using the values presented in Table 1 and Table 2.

Index Test Performance				
	IgM		IgG	
	*n*	% (95% CI)	*n*	% (95% CI)
Lot A				
Sensitivity	69/74	93.2 (85.1–97.1)	93/100	93.0 (86.3–96.6)
Specificity	200/200	100 (98.1–100)	199/200	99.5 (97.2–99.9)
PPV	69/69	100 (94.7–100)	93/94	98.9 (94.2–99.8)
NPV	200/205	97.6 (94.4–99.0)	199/206	96.6 (93.2–98.3)
Lot B				
Sensitivity	65/74	87.8 (78.5–93.5)	100/100	100 (96.3–100)
Specificity	200/200	100 (98.1–100)	199/200	99.5 (97.2–99.9)
PPV	65/65	100 (94.4–100)	100/101	99.0 (94.6–99.8)
NPV	200/209	95.7 (92.0–97.7)	199/199	100 (98.1–100)

The Wilson Score method was used to calculate confidence intervals for proportions. CI, confidence interval; IgG, immunoglobulin G; IgM, immunoglobulin M; PPV, positive predictive value; NPV, negative predictive value.

## Data Availability

Data available on request.
